# Whole heart free breathing phase sensitive inversion recovery MRI integrated with iterative self navigation for 100% scan efficiency; a first patient study

**DOI:** 10.1186/1532-429X-18-S1-O106

**Published:** 2016-01-27

**Authors:** Giulia Ginami, Davide Piccini, Simone Coppo, Tobias Rutz, Gabriele Bonanno, Gabriella Vincenti, Juerg Schwitter, Matthias Stuber

**Affiliations:** 1 Department of Radiology, University Hospital (CHUV) and University of Lausanne (UNIL), Lausanne, Switzerland; 2 Advanced Clinical Imaging Technology, Siemens Healthcare IM BM PI, Lausanne, Switzerland; 3 grid.8515.90000000104234662Department of Radiology, University Hospital (CHUV), Lausanne, Switzerland; 4 grid.8515.90000000104234662Division of Cardiology and Cardiac MR Center, University Hospital of Lausanne (CHUV), Lausanne, Switzerland; 5 grid.433220.4Center for Biomedical Imaging (CIBM), Lausanne, Switzerland

## Background

Phase Sensitive Inversion Recovery (PSIR) [1] allows for the visualization of myocardial scars using late gadolinium enhancement (LGE), ensuring robustness with respect to sequence timing. 3D whole-heart PSIR has been integrated with diaphragmatic navigator-gating (NAV) [2] to compensate for respiratory motion. However, both NAV and the need for two different datasets to be acquired (IR and reference) lead to a prohibitively long scanning time. Thus, integrating 1D respiratory Self-Navigation (SN) [3] with 3D-PSIR to obtain 100% scan efficiency is desirable. Unfortunately, signal and contrast variations between the IR and the reference dataset pose a major challenge. Here, we hypothesized that a recently introduced contrast independent iterative approach to 1D SN (IT-SN) [4] effectively suppresses respiratory motion in 3D-PSIR acquisitions.

## Methods

Data acquisition was first performed in a cohort of 8 patients to ascertain the successful integration of IT-SN with 3D whole-heart PSIR, after a regular 2D PSIR LGE examination. In a second cohort (7 patients), data were acquired using a similar protocol after slow infusion (0.2 mmol/kg, Gadobutrol) yet without preceding 2D PSIR LGE. In both cases, a prototype 3D radial SN bSSFP [5] was used on a 1.5T MRI scanner (Magnetom Aera, Siemens). Imaging parameters were: TR/TE 2.9/1.45 ms, FOV 220 mm^3^, resolution 1.4 mm^3^, RF excitation angle 115°(IR) and 8°(reference), Bandwidth 140 Hz/Px, fat saturation, TI 250-320 ms. IT-SN was used for respiratory motion correction. For the first cohort, uncorrected PSIR datasets were compared with PSIR datasets corrected with IT-SN, by computing endocardial border sharpness (EBS), sharpness of visible scars (%SS) [6], and by visual grading provided by two experts (grades ranging from 4, fully diagnostic, to 0, non-diagnostic). For the second cohort, IT-SN corrected PSIR images were compared with IT-SN corrected IR images to evaluate the efficacy of myocardial signal suppression. SNR of blood and myocardium were computed together with CNR between blood and myocardium.

## Results

**Figure 1:**
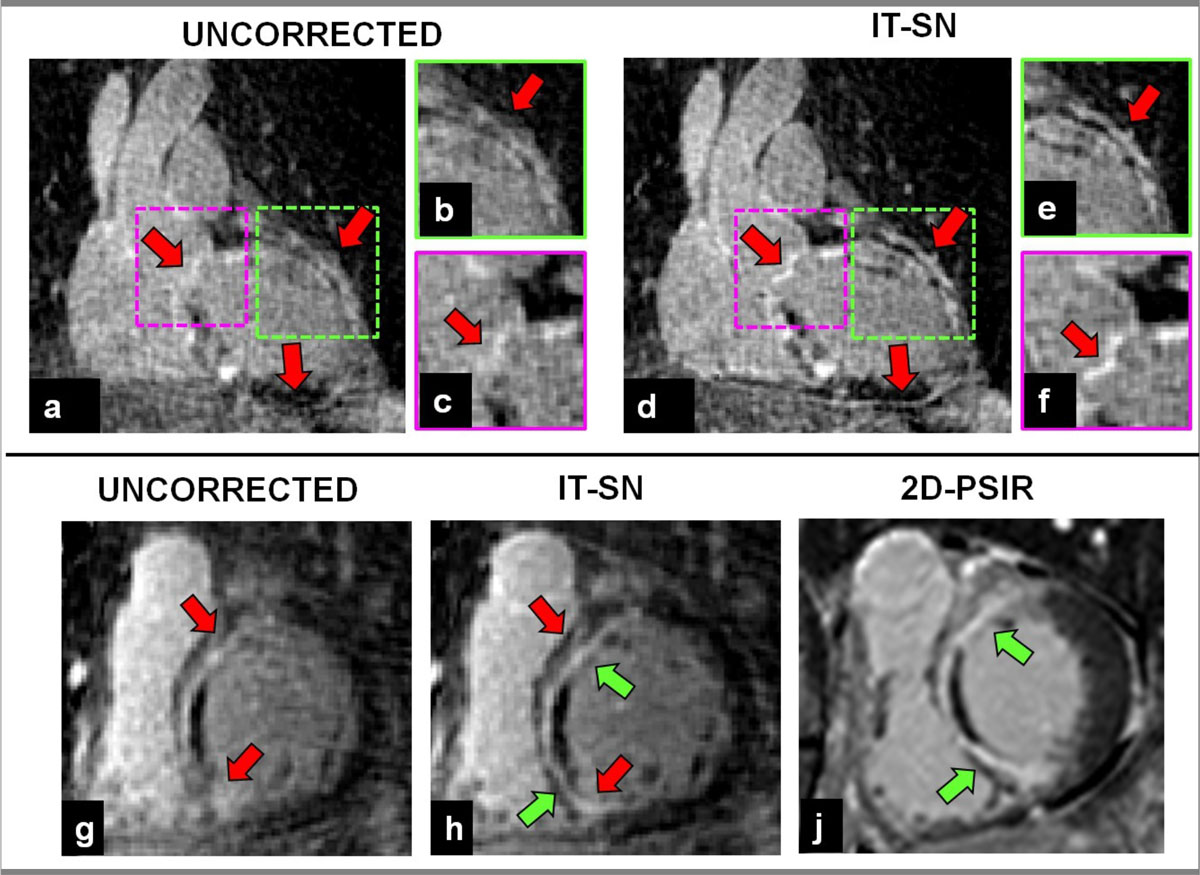
**Myocardial scars visualization in a patient with anterior myocardial infarction**. Sharp visualization of myocardial scars is obtained when IT-SN is applied (d) with respect to the uncorrected dataset (a), and as shown in the zoomed sections in b and e (red arrows). Furthermore, visualization of calcified structures, which are not detectable in the uncorrected dataset (c) become visible after the application of IT-SN (f). A comparison between uncorrected dataset (g) and IT-SN corrected dataset (h) is reported in short axis view; in this case, conventional 2D PSIR is also shown (j); again, image quality improves when IT-SN is applied (g and h, red arrows), and image quality of the 3D acquisition approaches that of the more conventional 2D PSIR method (h and j, green arrows).

**Figure 2:**
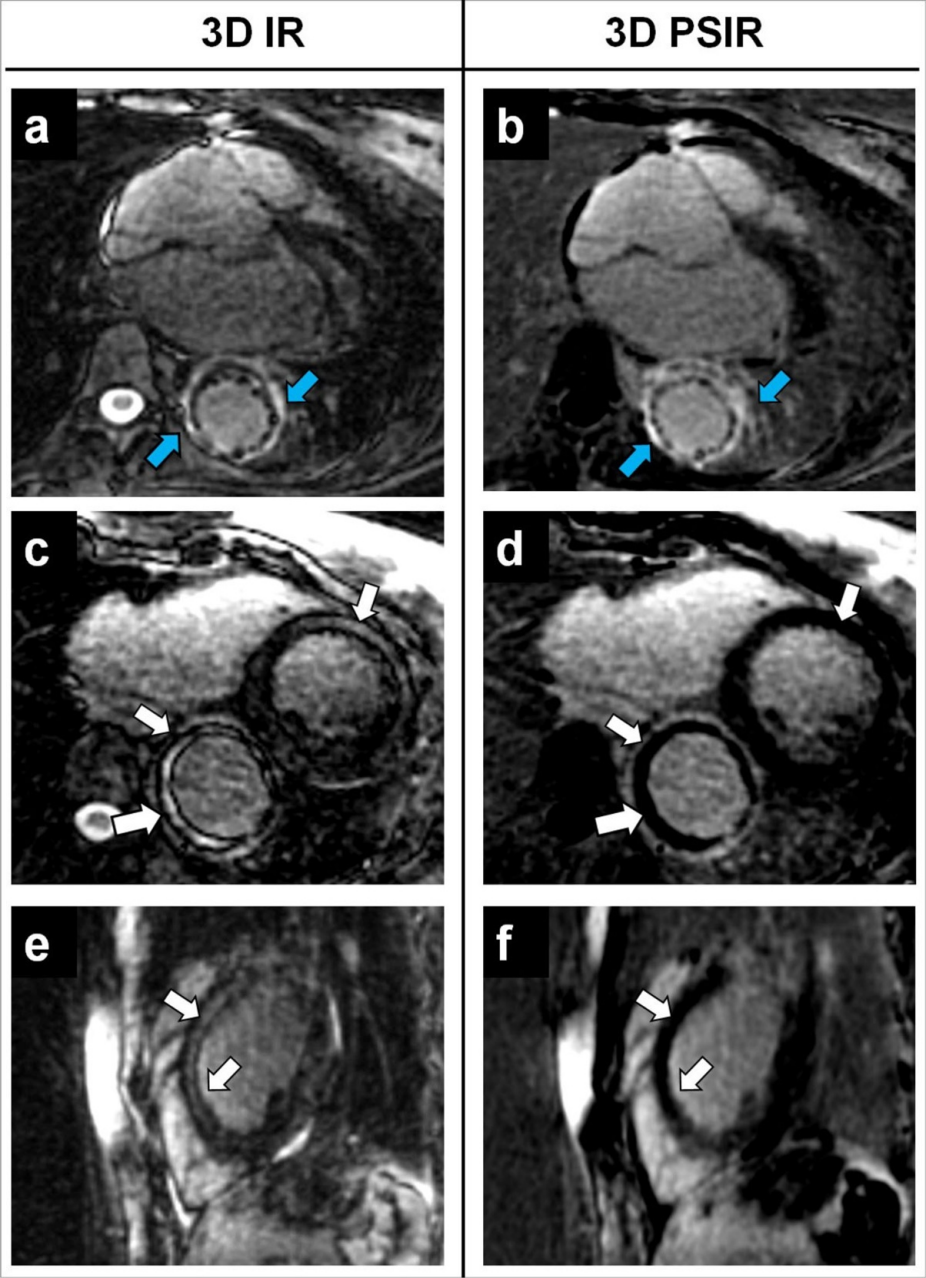
**Comparison between 3D IT-SN IR (a, c, e) and 3D IT-SN PSIR (b, d, f) in one patient with LGE findings in the descending aorta (blue arrow in a, b); here, and as indicated by the white arrows in c-d and e-f, nulling of healthy myocardial signal improves with the use of 3D PSIR, whereas myocardium does not appear to be properly suppressed in the case of 3D IR**.

For the first patient cohort, IT-SN improved image quality with respect to the uncorrected datasets (Fig 1), in terms of EBS (0.32 ± 0.14 mm^-1^ and 0.24 ± 1.30 mm^-1^, p < 0.05), %SS (14.0 ± 5.4 and 10.7 ± 7.4, p < 0.005), and visual grading (3.3 ± 0.8 and 2.7 ± 1.0, p < 0.05). In the second cohort, improved myocardial signal suppression was found when comparing IT-SN PSIR to IT-SN IR (Fig 2); SNR of the blood remained unchanged (%change 10.7 ± 28.0%, p = NS), SNR of the myocardium decreased (%change -31.5 ± 24.2, p < 0.05) and CNR improved (%change 64.3 ± 72.2, p < 0.02).

## Conclusions

IT-SN allows for free-breathing motion suppressed 3D whole-heart PSIR imaging with 100% scan efficiency. When compared to IT-SN IR, myocardial signal is more effectively suppressed, providing an improved delineation of scar tissue. Studies in a larger patient cohort are now warranted.
